# Post-transplant indolent T cell lymphoproliferative disorder in living donor liver transplantation: a case report

**DOI:** 10.1186/s40792-020-00904-y

**Published:** 2020-06-26

**Authors:** Ryoichi Goto, Norio Kawamura, Masaaki Watanabe, Yasuyuki Koshizuka, Souichi Shiratori, Momoko Ara, Shohei Honda, Tomoko Mitsuhashi, Yoshihiro Matsuno, Tsuyoshi Shimamura, Akinobu Taketomi

**Affiliations:** 1 Department of Gastroenterological Surgery I, Hokkaido University, Sapporo, Japan; 2 Department of Hematology, Hokkaido University, Sapporo, Japan; 3 Department of Surgical Pathology, Hokkaido University Hospital, Sapporo, Japan; 4 Division of Organ Transplantation, Hokkaido University Hospital, Sapporo, Japan

**Keywords:** Indolent T cell lymphoproliferative disorder, Post-transplant lymphoproliferative disorder, Living donor liver transplantation

## Abstract

**Background:**

Post-transplant lymphoproliferative disorder (PTLD) of T cell type has been rarely reported. Accurate diagnosis of this life-threatening rare form of PTLD is important for the treatment strategy.

**Case presentation:**

A 7-year-old boy had severe diarrhea and weight loss progressively at 7 years post-living donor liver transplantation (LDLT) for biliary atresia. Endoscopy in the gastrointestinal (GI) tract revealed multiple erosions and ulcer lesions with prominent intraepithelial lymphocytosis in the duodenum and terminal ileum. Immunohistochemical examination demonstrated that these accumulated lymphocytes mainly comprised small- to medium-sized T cells expressing CD3, CD4, CD5, CD7, and CD103, but lacking CD8, CD56, and Epstein-Barr virus-encoded small RNAs. In addition, T cell receptor β gene rearrangement was detected by polymerase chain reaction analysis. Comprehensively, the lesions were best interpreted as post-transplant indolent T cell lymphoproliferative disorder (LPD) of the intestine. Clinical remission was achieved by reducing the immunosuppressant.

**Conclusion:**

A rarely reported indolent type of T cell LPD in post-LDLT was diagnosed by direct inspection and histological investigation. Although the histological classification and therapeutic strategy for post-transplant indolent T cell LPD have not been established, reducing immunosuppression allowed complete remission in our case. To prevent the incidence of PTLD and de novo malignancy, developing a methodology to set a proper dose of immunosuppressant is required.

## Background

Post-transplant lymphoproliferative disorder (PTLD) is recognized as a heterogeneous morphologic feature ranging from non-destructive early lesions such as lymphoid hyperplasia to polymorphic or monomorphic lesions such as malignant lymphoma with a wide range of clinical manifestations. The most common phenotype of PTLD is B cell lymphoproliferative disorder (LPD) associated with Epstein-Barr virus (EBV). Recently, EBV-negative PTLD is frequently experienced in the clinical setting. It has been reported that the more frequent occurrence of EBV-negative PTLD was observed at later time points [1]. In addition, T cell PTLD, an extremely rare entity, occurs at greater than 5 years post-transplantation [2–5]. More frequent types of T cell PTLD have been reported to include peripheral T cell lymphoma, not otherwise specified, and hepatosplenic T cell lymphoma [2, 4, 5], although the T cell PTLD entity remains unclassified [6]. Herein, we report a case with a rare T cell PTLD, indolent T cell lymphoproliferative disease (LPD) in the gastrointestinal (GI) tract long after living donor liver transplantation (LDLT).

## Case presentation

**Fig. 1:**
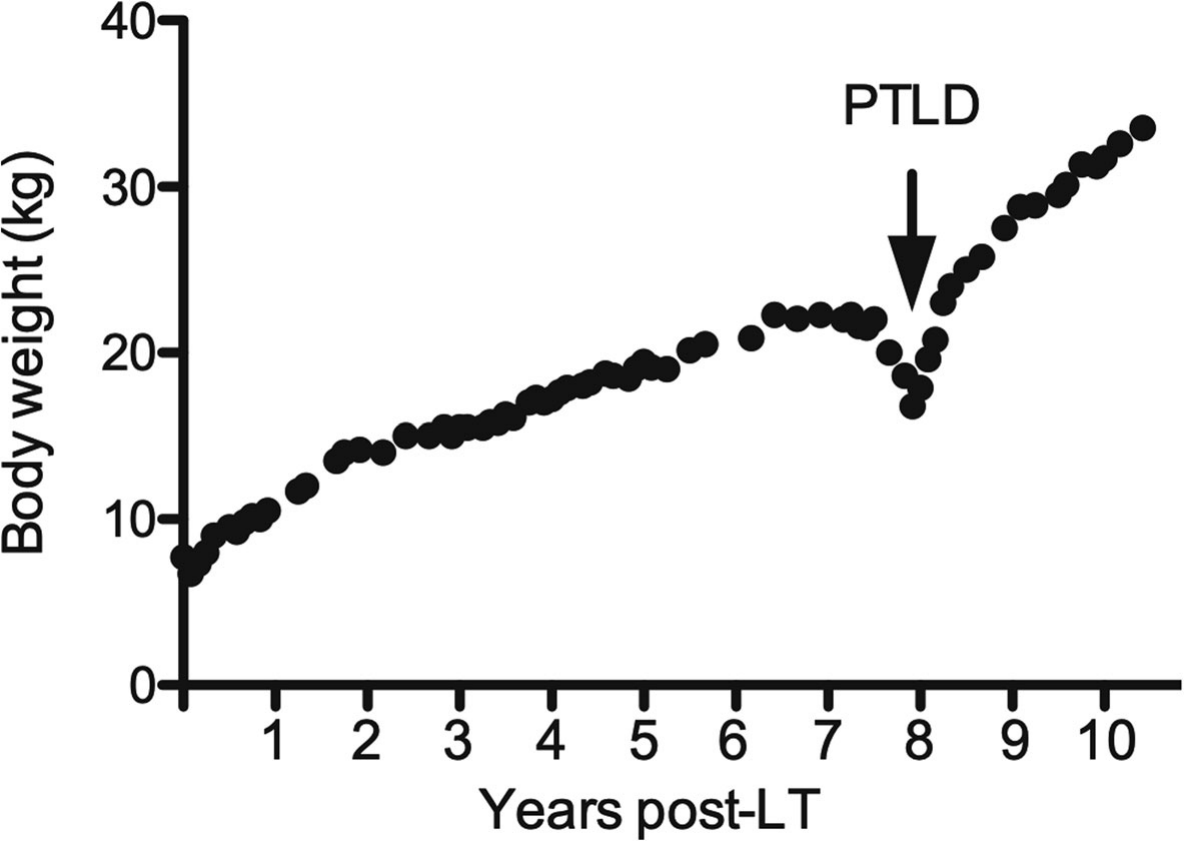
The body weight post-LT. The data of body weight post-LT were plotted. He had stopped gaining body weight 6 years post-LT and eventually lost body weight. The withdrawal of IS improved the clinical manifestations and recovered body weight gain

One day after birth, the patient had been diagnosed with meconium peritonitis and congenital obstruction of the small intestine, apple peel type. He received emergency laparotomy with a partial resection of the small intestine and reconstruction of the intestinal stoma 2 days after birth. He recovered a stoma 84 days after birth, when the length of remnant small intestine was determined to be about 80 cm. At 168 days after birth, liver function progressively got worse and a pathological examination of a liver biopsy specimen was diagnosed as biliary atresia (BA) with cholestatic decompensated liver cirrhosis. At 1 year old, he underwent LDLT for BA directly: no Kasai’s operation. His mother was the living donor. EBV status of the recipient was seronegative, but the donor was seropositive, so we carefully monitored the measurement of EBV-DNA copies in serum. The immunosuppressant (IS) protocol of basiliximab (10 mg/body on days 1 and 4 post-LT), tacrolimus (oral dose of 0.6 mg/body started on day 3 at the targeted trough level around 10–12 ng/ml; to achieve the target trough level, continuous intravenous infusion was applied on day 17 post-LT), methylprednisolone (0.5 mg/kg [4 mg/body] started on day 1 and discontinued on day 14 post-LT due to steatotic liver graft), and mycophenolate mofetil (10 mg/kg/day [80 mg/body] started on day 1 and discontinued at 6 weeks post-LT due to CMV infection; to reach the appropriate trough level, dose escalation to 50 mg/kg was required) was applied post-transplantation according to our institutional IS protocol at that time. He had an episode of moderate acute cellular rejection, which was treated with pulsed intravenous methylprednisolone 9 days post-transplantation. Later, he grew steadily (Fig. 1) and the IS protocol was changed to a monotherapy of tacrolimus administered at a high dose (9 mg of Graceptor®, a once-daily tacrolimus extended-release formulation) to maintain a trough level of the drug (5–6 ng/ml), probably because of malabsorption caused by the shortened bowel.

**Fig. 2:**
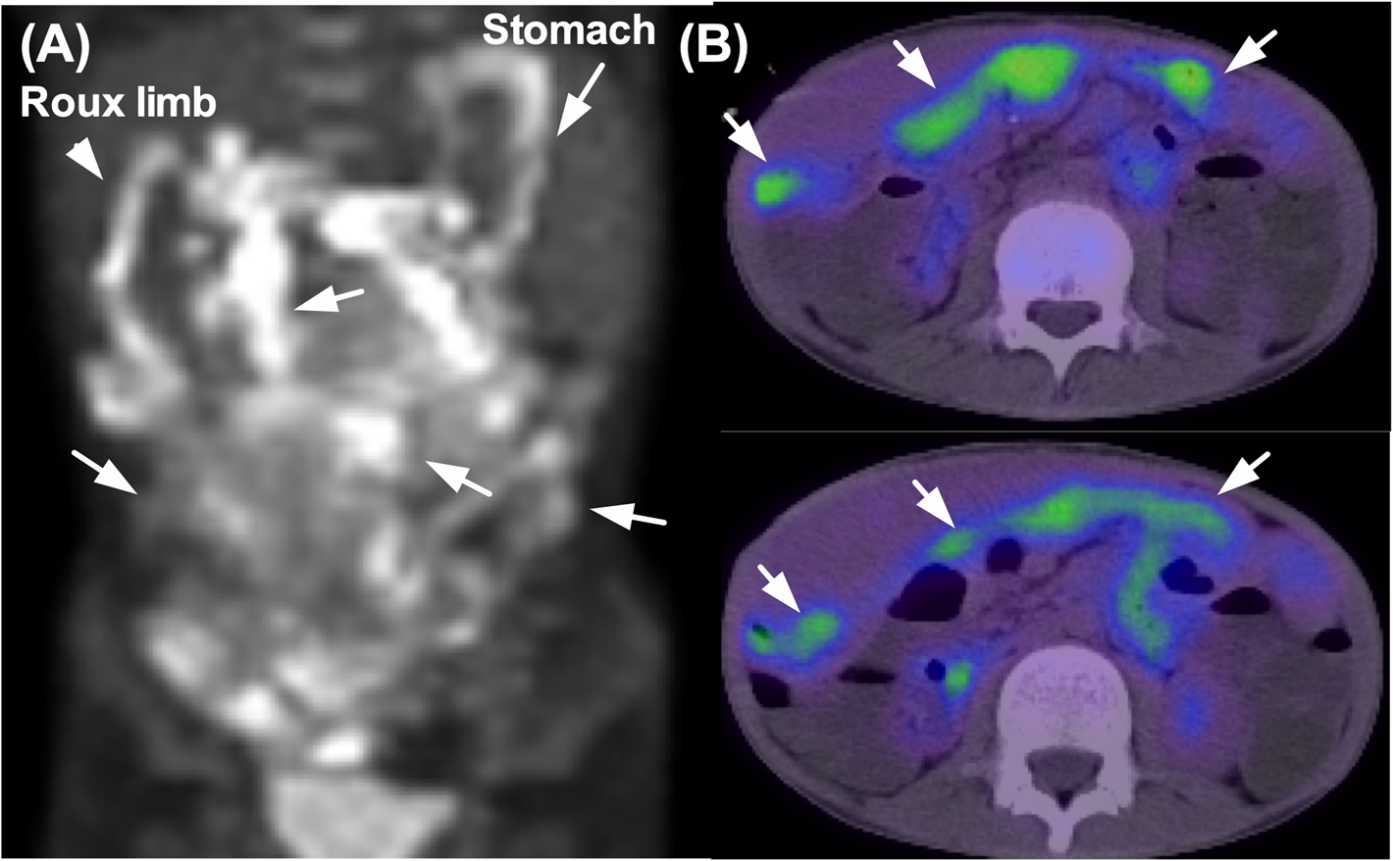
PET-CT: PET-CT revealed a wide range of the small intestine increased FDG activity. **a** The image of the whole abdomen in FDG activity. **a**, **b** The arrowhead indicates the jejunum of the Roux limb. The arrows indicate the stomach and small intestine with increased FDG activity

**Fig. 3:**
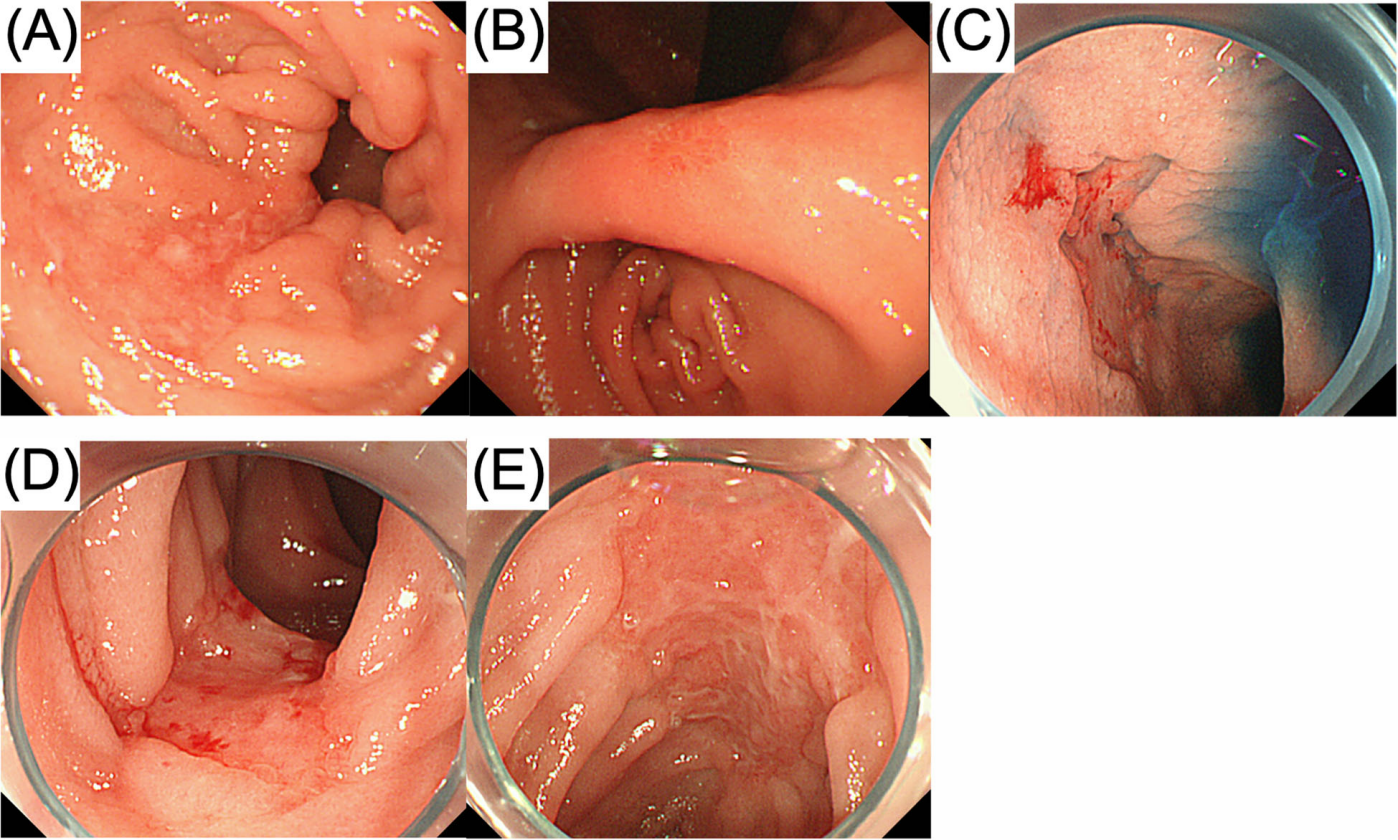
Findings of the GI tract. **a**, **b** Upper GI endoscopy revealed gastric erosions under inflamed mucosa (**a**) and duodenal superficial ulcers (**b**). **c**–**e** Colonoscopy with ileoscopy detected multiple ulcers in the terminal ileum

**Fig. 4:**
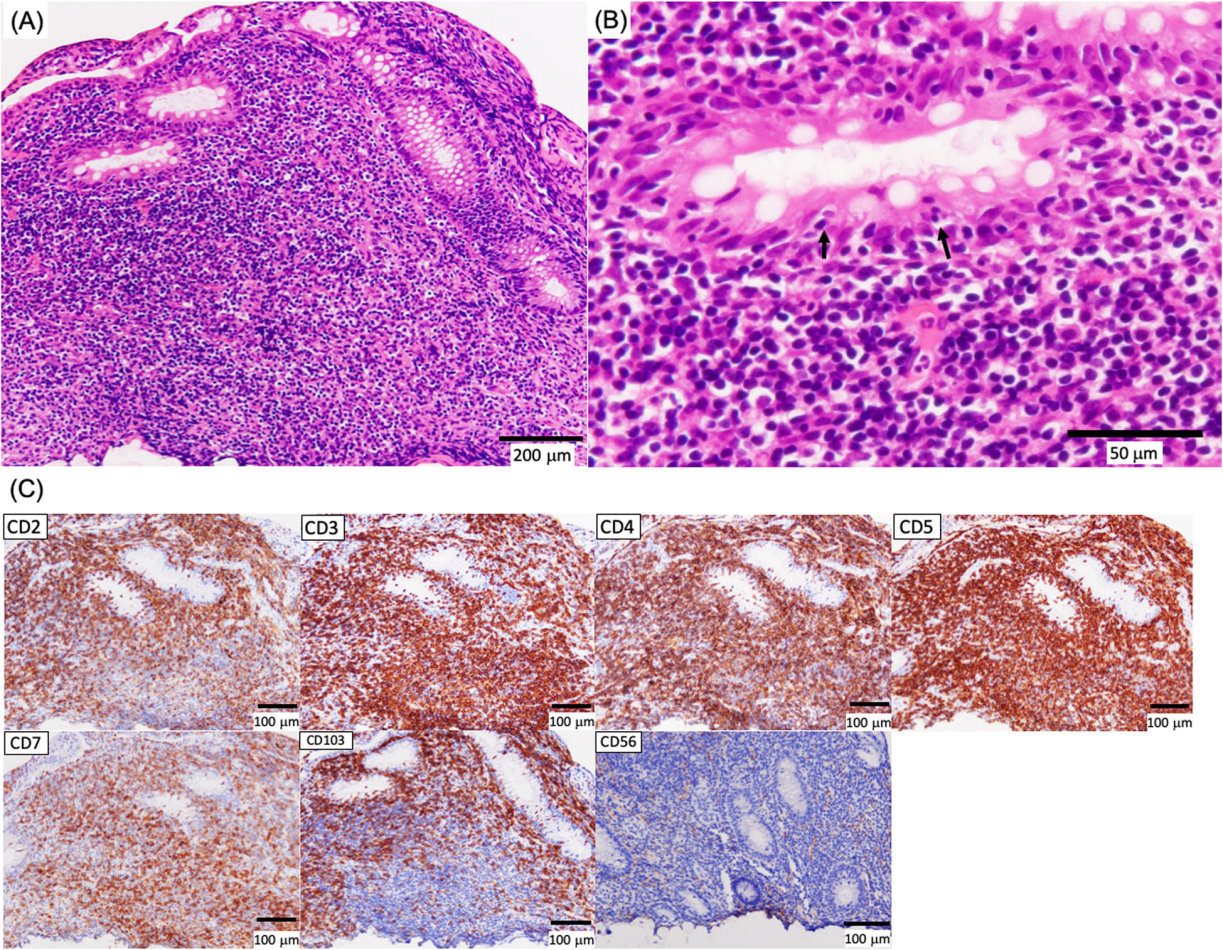
Histopathological examination in the tissue biopsy of the GI tract. **a**, **b** Ulceration in the ileum. **a** The lesion surrounded by a dense lymphocyte infiltration around the crypts. **b** Most infiltrating lymphocytes were small to medium size without significant nuclear atypia. Cryptitis was found in areas as shown by the arrows. **c** Immunohistochemically, the lymphocytes express CD2, CD3, CD4, CD5, CD7, and CD103 and lack CD56 expression

When the patient was 7 years old, he had seborrheic diarrhea and weight loss without appetite loss (Fig. 1). His body weight fell significantly to lower than the − 2 SD for his age (Fig. 1). A blood examination revealed anemia and hypoalbuminemia. No abnormal data were shown in a hormonal examination, and the EBV-DNA copies in his serum were 1500. It was likely that his malabsorption was associated with short bowel syndrome. However, nutritional support, including central venous parenteral nutrition, could not improve his status. Abdominal US examination showed some gasses within the portal vein, a thin intestinal wall, and a dilatation of the sigmoid colon and rectum. A CT examination demonstrated bowel dilatation with fluid and enlarged mesenteric lymph nodes. A positron emission tomography (PET)-CT scan displayed high intensity FDG uptake in the whole intestine (Fig. 2). Gastroduodenal endoscopy revealed gastric erosion under inflamed mucosa (Fig. 3a) and a duodenal superficial ulcer (Fig. 3b). In addition, colonoscopy with ileoscopy detected multiple ulcers in the terminal ileum (Fig. 3c–e). Tissue biopsy revealed dense mononuclear cell infiltration in the lamina propria, partly forming intracryptic and intraepithelial lymphocytosis (Fig. 4a). Non-specific granulation tissue infiltrated by mixed inflammatory cells was also associated with erosions and ulcers. These mononuclear cells were rather monomorphic small- to medium-sized lymphocytes with minimal nuclear atypia. Paraffin section immunohistochemical analysis showed that the lymphocytes were almost uniformly positive for CD2, CD3, CD4, CD5, CD7, and CD103, but negative for CD20, CD8, and CD56 (Fig. 4c). In addition, immunostaining for cytotoxic molecules (granzyme B, TIA-1, perforin) was negative (data not shown). None of the mononuclear cells showed positivity for EBV-encoded small RNAs by in situ hybridization (EBER-ISH) or immunoreactive EBV nuclear antigen 2 (EBNA2). The Ki-67 labeling index was less than approximately 10%. Polymerase chain reaction (PCR) analysis using formalin-fixed paraffin-embedded tissue for T cell receptor (TCR) β demonstrated clonal gene rearrangement. In total, these pathological findings were interpreted as a post-transplant lymphoproliferative disorder, most likely indolent T cell lymphoproliferative disorder of the GI tract. Our institution’s multidisciplinary tumor board decided to reduce the IS to 2 mg of Graceptor® with a lower trough level. Firstly, target trough level was less than 5 ng/ml, and later, we decided it according to the clinical course and histological finding of liver biopsy. Eventually, the daily dose of tacrolimus was 0.4 mg with a trough level below measurement sensitivity.

Two months after IS reduction, endoscopic findings showed that multiple ulcers and erosions in the duodenum and terminal ileum were remnant; however, the CD3^+^ lymphocyte infiltration was slightly reduced in the lesions. TCR Jβ1 gene rearrangement could not be proven in frozen specimens by southern blotting analysis. EBV-DNA was gradually reduced from 1500 copies to 1000 copies at 1 month, 990 copies at 2 months, and 340 copies at 3 months after IS reduction. The patient’s nutritional status was dramatically improved, and body weight was recovered to 24 kg 5 months after IS reduction, which is equivalent to 150% of his body weight at IS reduction. Upper GI endoscopy and colonoscopy finally revealed a complete recovery; neither ulceration nor erosion was apparent in the GI tract at 9 months and 1 year after IS reduction.

## Discussion

PTLD is difficult to notice because the laboratory surveillance protocols in the early stage and PTLD prevention methods have not been established, in particular in EBV-negative entities. In our case, the patient received high doses of Graceptor® to maintain the blood concentration of tacrolimus for 7 years. In some patients, the EBV viral load sometimes lets us reduce the IS, but not in this case. The high intensity of IS for years was probably associated with the occurrence of lymphoproliferative disorder. A previous study showed that calcineurin inhibitors increased the risk of PTLD [7]. Indeed, the reduction of tacrolimus led to improvement of the clinical manifestation in our case. PTLD is a heterogeneous disease ranging from polymorphic hyperplasia to monomorphic lymphoproliferative disorder or malignant lymphoma. Usually, the polymorphic type is reversible on reduction of IS; however, late onset, monomorphic, or EBV-negative lymphomas are often unresponsive to IS reduction or discontinuation [7]. In our case, proliferating lymphocytes were monomorphic, but showed only subtle nuclear atypia and a low Ki-67 labeling index in addition to being the T cell phenotype. These unusual pathologic characteristics of this type of lesion may allow for only IS reduction in achieving a complete remission.

It has been reported that risk factors of PTLD are EBV seronegativity of the recipient or the pediatric recipient [7], being an older adult donor, intensity of IS, and first year post-transplantation [8]. PTLD associated with EBV infection occurs in the first to second year post-transplantation. Although early year post-transplantation is a risk for PTLD [7], a nationwide study in France with long-term follow-up showed that, though rare, an increased incidence of PTLD was observed even at 10 years post-transplantation [8]. The occurrence of EBV-negative PTLD has been increasing between 7 and 10 years after transplantation. The French study also showed that GI tract PTLD was increased dramatically from 6 years post-transplantation, where monomorphic type was more frequent in digestive PTLD [8]. In addition, in the kidney transplant registry, EBV-negative PTLD occurs long (> 5 years) after transplantation [9]. In our case, EBV infection was not proven with intraepithelial lymphocytosis in the GI tract lesion; PTLD occurred 7 years after liver transplantation. That it was EBV-negative and was a GI tract lesion corresponded to a previous report.

In terms of T cell PTLDs, a previous review reported that it was uncommon, comprising 5 to 15% of all PTLD [2, 5]. It occurs in all ages (people from 2 to 75 years old) and at later time points (median 5.5 years post-transplantation) [5]. The majority of T cell PTLDs occur following kidney transplantation (66–69%), and 5–11% are seen post-liver transplantation [2, 5]. In addition, the majority of T cell PTLDs are not associated with the EBV load [2, 10–12]. The GI tract is a frequent site of PTLDs comprising 15% of all T cell PTLDs developing in the extranodal sites [5]. In this case, a low level of EBV-DNA copies in serum was detected. However, none of the mononuclear cell-infected EBV was observed by double EBER-ISH and immunohistochemical assay. Also, T cells do not usually express EBV receptor CD21 [2]. Therefore, T cell lymphoproliferative disease in this case may not be pathologically associated with EBV infection. During improving the clinical course, gradual decline in EBV-DNA copies in serum might be affected by his immune status.

In general, there are two major categories of intestinal T cell lymphomas: enteropathy-associated T cell lymphoma (EATL), formerly called type I EATL, and monomorphic epitheliotropic intestinal T cell lymphoma (MEITL), formerly type II EATL. In contrast to these two lymphoma types, which both show aggressive clinical behavior, another type with an indolent clinical course was recognized and described in the 2016 revision of the 4th edition of the WHO classification [13] as a provisional entity. This type, indolent T cell LPD of the GI tract, has a unique feature characterized by low-grade histology with small lymphocytic proliferation [14, 15]. However, the precise histopathologic or phenotypic criteria remain to be well established [15]. In our case, CD4^+^ T cells predominantly infiltrated the lesion. Indolent CD4^+^ T cell LPD has been rarely reported [16, 17]. A review of indolent CD4^+^ T cell LPD in 2017 showed that just 27 cases have been described until that time [17]. The median age of patients was 51.5 (22–68) years old. The clinical symptoms of these LPDs of the GI tract included chronic diarrhea and weight loss [17, 18]. Small bowel involvement may be associated with diarrhea [17]. Usually, the disease was localized to the GI tact for a long duration without extra lesions. Histopathological examination showed that scattered plasma cells and eosinophils were seen in the superficial lamina propria. Granulomas were occasionally reported in the small bowel mucosa, and the mitotic activity was low [17]. Immunophenotypical analysis of indolent CD4^+^ T cell LPD demonstrated that it was positive for CD3 and CD4 in all cases and negative for CD5, CD7, and CD56 in 33%, 50%, and 100% of cases, respectively [17]. In our case, the lymphocytes expressed CD4, CD3, CD5, CD7, and CD103. CD103 expression is one of the key phenotypes of MEITL, but is not usually expressed in indolent CD4^+^ T cell lymphoproliferative disease [17]. However, the present case is clearly distinguished from MEITL, not only because of its indolent clinical course, but also owing to the lack of immunohistochemical expression of CD8 or cytotoxic molecules such as granzyme B, TIA-1, or perforin. Of note, our case demonstrated a clonal TCRβ gene rearrangement corresponding to a previous report on an indolent CD4^+^ T cell LPD [17]. A variety of chemotherapies were applied for indolent CD4^+^ T cell LPD. Although 83% of patients demonstrated persistent disease and 13% of patients died, the risk for disease transformation appears low [17, 18] so a careful “watch and see” approach was recommended [17]. However, persistent indolent T cell LPD over a long term (4.6–25 years) has been reported [18]. Importantly, in cases of post-transplantation and in immunodeficient states, indolent T cell LPD as a PTLD has been rarely reported [19]. To our best knowledge, our case is the second report of indolent T cell LPD of the GI tract after solid organ transplantation [20]. A careful follow-up is necessary although complete improvement and clinical remission has been achieved in our case.

## Conclusions

We had a very rare case of PTLD, indolent T cell LPD. Because the number of long-term post-transplantation patients has been increasing, the understanding of EBV-negative, GI tract LPD entities and their risks is important for patient care in organ transplantation.

## Data Availability

Not applicable

## Abbreviations

BA: Biliary atresia
EATL: Enteropathy-associated T cell lymphoma
EBV: Epstein-Barr virus
EBER-ISH: EBV-encoded small RNA in situ hybridization
GI: Gastrointestinal
IS: Immunosuppressants
LDLT: Living donor liver transplantation
LPD: Lymphoproliferative disorder
MEITL: Monomorphic epitheliotropic intestinal T cell lymphoma
MMF: Mycophenolate mofetil
mPSL: Methylprednisolone
PET: Positron emission tomography
PTLD: Post-transplant lymphoproliferative disorder
